# Identifying Stage II Colorectal Cancer Recurrence Associated Genes by Microarray Meta-Analysis and Building Predictive Models with Machine Learning Algorithms

**DOI:** 10.1155/2021/6657397

**Published:** 2021-02-10

**Authors:** Wei Lu, Xiang Pan, Siqi Dai, Dongliang Fu, Maxwell Hwang, Yingshuang Zhu, Lina Zhang, Jingsun Wei, Xiangxing Kong, Jun Li, Qian Xiao, Kefeng Ding

**Affiliations:** ^1^Department of Colorectal Surgery and Oncology, Key Laboratory of Cancer Prevention and Intervention, Ministry of Education, The Second Affiliated Hospital, Zhejiang University School of Medicine, Hangzhou, Zhejiang, China; ^2^Cancer Center, Zhejiang University, Hangzhou, China

## Abstract

**Background:**

Stage II colorectal cancer patients had heterogeneous prognosis, and patients with recurrent events had poor survival. In this study, we aimed to identify stage II colorectal cancer recurrence associated genes by microarray meta-analysis and build predictive models to stratify patients' recurrence-free survival.

**Methods:**

We searched the GEO database to retrieve eligible microarray datasets. The microarray meta-analysis was used to identify universal recurrence associated genes. Total samples were randomly divided into the training set and the test set. Two survival models (lasso Cox model and random survival forest model) were trained in the training set, and AUC values of the time-dependent receiver operating characteristic (ROC) curves were calculated. Survival analysis was performed to determine whether there was significant difference between the predicted high and low risk groups in the test set.

**Results:**

Six datasets containing 651 stage II colorectal cancer patients were included in this study. The *microarray* meta-analysis identified 479 recurrence associated genes. KEGG and GO enrichment analysis showed that G protein-coupled glutamate receptor binding and Hedgehog signaling were significantly enriched. AUC values of the lasso Cox model and the random survival forest model were 0.815 and 0.993 at 60 months, respectively. In addition, the random survival forest model demonstrated that the effects of gene expression on the recurrence-free survival probability were nonlinear. According to the risk scores computed by the random survival forest model, the high risk group had significantly higher recurrence risk than the low risk group (HR = 1.824, 95% CI: 1.079–3.084, *p* = 0.025).

**Conclusions:**

We identified 479 stage II colorectal cancer recurrence associated genes by microarray meta-analysis. The random survival forest model which was based on the recurrence associated gene signature could strongly predict the recurrence risk of stage II colorectal cancer patients.

## 1. Introduction

Colorectal cancer is the third most common cancer and also a leading cause of cancer mortality worldwide [1]. About half of colorectal cancer patients presented with early stage diseases (stage I–II), and about 25% of patients presented with locally advanced stage diseases (stage III), yet the rest of patients had distant metastasis (stage IV) [2, 3]. Surgical resection and adjuvant chemotherapy were the most common treatments for colorectal cancer and helped patients gain significant survival benefit, especially for those early stage patients [4, 5]. However, more than 20% of colorectal cancer patients suffered from disease recurrence after primary tumor resection, with the majority of them having metastatic recurrence [6]. It was noteworthy that most of colorectal cancer related deaths were attributed to disease recurrence [7]. In addition, recurrence-free survival time of colorectal cancer patients was strongly associated with overall survival time [8]. Hence there is an urgent need to discover new prognostic markers and develop predictive models for colorectal cancer recurrence.

Traditionally, it was believed that the prognosis of colorectal cancer was largely determined by the TNM stage at the time of diagnosis. However, there was a survival paradox that stage IIB patients had higher recurrence rate and worse survival than stage IIIA patients [9, 10]. For stage II patients, although pT4 stage and mismatch repair deficiency (dMMR/MSI-H) could stratify patients into higher or lower recurrence risk groups, respectively, early recurrence still occurred among those alleged low recurrence risk patients [11–13]. Towards the need for personalized cancer care, it was necessary to build predictive models, especially for stage II colorectal cancer patients.

With the rapid development of high throughput technology, identifying cancer molecular subtypes and building molecular prognostic models *gradually* came to reality. Guinney et al. created the colorectal cancer consensus molecular subtypes, which consisted of four subtypes with distinguishing biological behaviors and clinical characteristics [14]. In terms of colorectal cancer recurrence, some researchers have made great efforts to develop predictive models based on cancer molecular characteristics. For example, Dai et al. built the lasso Cox model based on 15 gene expression values to identify stage I–III colon cancer patients with high recurrence risk [15]. Agesen et al.developed a classifier based on 13 gene expression values, which was also known as the ColoGuideEx, to predict stage II colorectal cancer patients' recurrence-free survival [16]. However, there was still room for improvement for current models. First, most of the models were based on single or a few datasets and were not limited to stage II patients, and so far no universal recurrence associated genes in stage II colorectal cancer were recognized. In addition, the methods of building and training models were mainly linear models, such as Cox regression or lasso Cox regression, yet the relationship between specific gene expression value and cancer recurrence risk might be nonlinear, which would probably reduce the prediction accuracy of models.

In this study, we included microarray data of 651 stage II colorectal cancer patients and identified a group of recurrence associated genes by microarray meta-analysis. We further performed the feature selection process and built predictive models with different machine learning algorithms, such as lasso Cox model and random survival forest model, and we found that the random survival forest model based on universal recurrence associated gene signature could strongly predict the recurrence risk of stage II colorectal cancer patients.

## 2. Materials and Methods

### 2.1. Searching and Screening Datasets

We searched the GEO database (http://www.ncbi.nlm.nih.gov/geo/) to retrieve potentially eligible microarray datasets. The search strategy was as follows: (“colorectal cancer” OR “colon cancer” OR “rectal cancer”) AND (“Expression profiling by array”). First, we filtered datasets by screening titles and abstracts. Then, we further screened datasets according to the inclusion and exclusion criteria. The inclusion criteria were as follows: patients had stage II colorectal cancer with recurrence-free survival follow-up data; experimental platforms were gene expression array. The exclusion criteria were as follows: dataset sample sizes were less than 40; datasets only provided whether patients had *recurrent* events without follow-up time. The following information of eligible datasets was extracted: series accession number, microarray experimental platform, sample size, recurrence-free survival follow-up duration, and recurrence rate.

### 2.2. Microarray Data Preprocessing and Quality Control

Raw data and platform sets of each dataset were downloaded from the GEO database (http://ftp.ncbi.nih.gov/geo/series/). Raw data (CEL files) of each dataset were read into R using the “oligo” package (version 1.42.0), and then background correction, normalization, and summarization were performed using the RMA algorithm [17]. Microarray data quality control was assessed by hierarchical clustering based on the distance between samples in Pearson's correlation matrices, and the height cut-off value was set to 0.20 to recognize potential outliers [18], which were removed before further analysis.

### 2.3. Microarray Meta-Analysis

To identify universal recurrence associated genes, we performed microarray meta-analysis following the guidelines proposed by Ramasamy et al. [19]. Universal recurrence associated genes were identified using the “MetaDE” package (version 1.0.5) in R based on the recurrence-free survival and censored data [20]. The log-rank test was used to calculate *p* values of each gene in individual datasets, and then *p* values were pooled via the minP method [20]. On account of multiple statistical tests, the false discovery rate (FDR) controlling procedures were performed via the Benjamini–Hochberg method, with the cut-off value of 0.10 to select candidate genes for further analysis [21].

### 2.4. Enrichment Analysis and Protein-Protein Interaction Network Analysis

To uncover the biological characteristics of the selected genes, we used the Metascape tool (http://metascape.org) to perform KEGG and GO terms enrichment analysis, which consisted of pathway and function set enrichment analysis [22]. For universal recurrence associated genes identified by microarray meta-analysis, genes included in the microarray meta-analysis were set as the enrichment background, then we calculated *p* values of enriched KEGG and GO terms based on the hypergeometric distribution of expected terms, and finally enrichment bar plots were displayed and colored by *p* values. Protein-protein interaction network of the selected genes was performed using STRING database (http://string-db.org/) and visualized using Cytoscape software [23]. Proteins which interacted with more than 10 other proteins were considered as potential hub genes.

### 2.5. Batch Effect Normalization, Correlation Analysis, and Principal Component Analysis

Expression profiles of universal recurrence associated genes were extracted from the included datasets. To minimize microarray batch effect, we adopted ComBat to perform batch effect normalization, and we selected GSE39582 as the reference batch due to its largest sample size. Correlation analysis was performed by calculating the Pearson correlation coefficient of universal recurrence associated genes, and it was visualized using the “corrplot” package (version 0.84) [24]. Based on the gene expression profiles, principal component analysis was conducted using “stats” package (version 3.4.2), and the first 3 principal components were displayed in scatter plots [25]. In addition, proportions of variance explained by principal components were also plotted using the “ggplot2” package (version 2.2.1) [26].

### 2.6. Lasso Cox Model and Random Survival Forest Model

We randomly divided total samples into the training set (60%) and the test set (40%), and then lasso Cox model and random survival forest model were trained. The lasso Cox model was built and trained using the “glmnet” package [27], which was the penalized Cox regression model with the least absolute shrinkage and selection operator method, and it was generally used in high dimensional data and could help with the variable selection procedure [28, 29]. By placing a constraint on the absolute value of regression coefficients, the lasso Cox model forced numerous regression coefficients to become smaller or exactly zero [29]. Partial likelihood deviance was selected as the loss function, and the penalty parameter *λ* was determined through 20-fold cross validation to reach the minimal loss function value [29]. Regression coefficients of genes were calculated with the optimal *λ* value, and recurrence risk scores of patients were then summarized based on the expression level of genes and their regression coefficients accordingly.

The random survival forest model consisted of plenty survival trees, which were trained on bootstrap samples of the training set. Each survival tree had one root node that would branch out into two child nodes. The node-splitting rules were as follows (Figure S1): A given number of variables were randomly selected as candidate variables in each root node, then the optimized cut-off values of candidate variables were generated to maximize the survival difference of child nodes, and only the most discriminative candidate variable would become the node-splitting variable. Iteratively, child nodes became parent nodes, and the above node-splitting processes were repeated until the number of samples in the terminal node was less than the prespecified number [30].

The random survival model is specially suitable for dealing with high dimensional data with survival outcomes, and it could assess the nonlinear effect and importance of variables [31]. In this study, tuning parameters, such as the prespecified number of samples in the terminal node (node size) and the number of candidate variables randomly selected in each parent node (mtry), were optimized by grid search to minimize the out-of-bag (OOB) error. In addition, the random survival forest model was an efficient tool for variable selection. The model used minimal depths to reflect the priority of variables being selected as node-splitting variables, and smaller minimal depths indicated greater importance of variables (Figure S1). We ranked variables according to the minimal depths and then filtered variables above the cut-off value [30]. Models were trained once again with variables below the cut-off value until no variables were above the cut-off value. Marginal effects of variables on the recurrence-free survival probability were also displayed. Finally, with the trained random survival forest model, recurrence risk scores of patients were calculated using the “predict” function of the “stats” package [25]. The random survival forest model was built and trained utilizing the “randomForestSRC” package [30].

### 2.7. Statistical Analysis

Time-dependent receiver operating characteristic (ROC) curve analysis was performed with the “timeROC” package (version 0.3) to assess the prognostic value of trained models, and a higher area under the ROC curve (AUC) value indicated better performance [32, 33]. In addition, Youden indexes (sensitivity + specificity − 1) of risk score cut-off values were calculated, and the optimized cut-off value was determined *when* Youden index reached the maximum. The concordance index (Harrell's C-index) was calculated to assess model fitness, which ranged from 0.5 (indicating random prediction) to 1 (indicating perfect prediction) [30]. The Mann–Whitney test was adopted to examine whether predicted recurrence risk scores were statistically significant when comparing the nonrecurrent group and the *recurrent* group. Kaplan–Meier survival curves were displayed using the GraphPad Prism 6 software, and the log-rank test was performed to find whether survival difference between groups was statistically significant. The packages used in the current study were all under the R environment (version 3.4.2) [25]. A two-tailed *p* value less than 0.05 showed statistical significance unless it was specified.

## 3. Results

### 3.1. Characteristics of the Included Datasets

**Figure 1:**
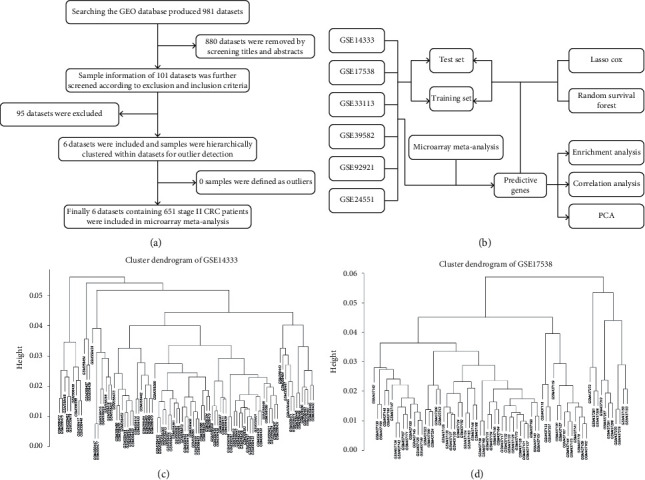
(a) Flow diagram of microarray datasets screening and selection. (b) Flow diagram of microarray meta-analysis and subsequent machine learning models. (c) Cluster dendrogram of the GSE14333 dataset. (d) Cluster dendrograms of the GSE17538 dataset. Another 4 cluster dendrograms of the included datasets were not shown.

As shown in Figures 1(a) and 1(b), we searched the GEO database and exported a total of 981 datasets; then, 880 datasets were removed by screening titles and abstracts. Next, 95 datasets were excluded according to the inclusion and the exclusion criteria; then, the remaining 6 datasets were included, and samples were hierarchically clustered for outliers detection, yet no samples were designated as outliers (Figures 1(c) and 1(d)). Finally, 6 datasets containing 651 stage II colorectal cancer patients were included in the following analysis.

The majority of the datasets adopted the GPL570 microarray platform (Affymetrix Human Genome U133 Plus 2.0 Array), and 1 dataset adopted the GPL5175 microarray platform (Affymetrix Human Exon 1.0 ST Array), as shown in Table 1. GSE39582 had the largest sample size of 267, while GSE92921 had the smallest sample size of 43, and the median recurrence-free survival follow-up duration varied from 37.31 to 74.63 months.

### 3.2. Identifying Universal Stage II Colorectal Cancer Recurrence Associated Genes

Based on recurrence-free survival and censored data, we first performed log-rank tests in individual datasets to calculate *p* values of each gene, which were then pooled via the minP microarray meta-analysis method [20]. The FDR controlling procedure was performed to solve the issue of multiple statistical tests, and a total of 479 significant genes were below the FDR cut-off value, as shown in Table S1.

### 3.3. Enrichment Analysis, Correlation Analysis, and Principal Component Analysis

**Figure 2:**
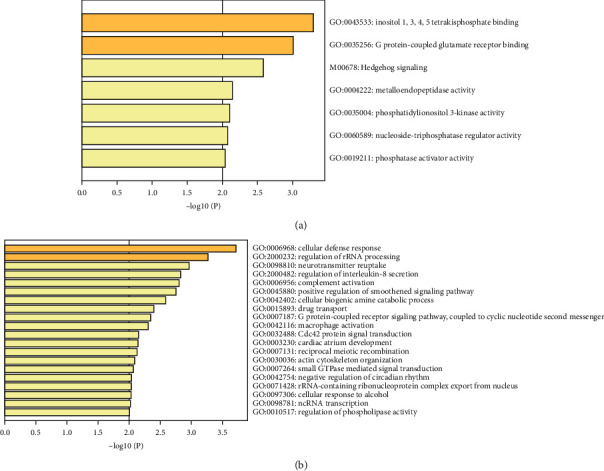
Bar plots of the enriched GO and KEGG. (a) Functional set and (b) pathway terms. The *x*-axis was log-transformed. Bars are colored by *p* values, and a darker color indicates smaller *p* values.

To further explore the function of universal stage II colorectal cancer recurrence associated genes, we performed the KEGG and GO terms enrichment analysis (Figure 2). Top function set terms were inositol 1,3,4,5-tetrakisphosphate binding, G protein-coupled glutamate receptor binding, and Hedgehog signaling, while top pathway terms were cellular defense response, regulation of rRNA processing, and neurotransmitter reuptake. Interestingly, some previous studies have reported the correlation of Hedgehog signaling and colorectal cancer recurrence [34]. In addition, dysregulation of small GTPase family proteins and their regulators was shown to have prognostic value for colorectal cancer recurrence [35–37]. Protein-protein interaction network of 479 significant genes is shown in Figure S2. We found that there were intensive protein-protein interaction pairs, and 3 potential hub genes (POLR2B, GNB2, and GNG2) were identified, which suggested that universal recurrence associated genes may cooperate together to have an impact on the recurrence of stage II colorectal cancer patients.

**Figure 3:**
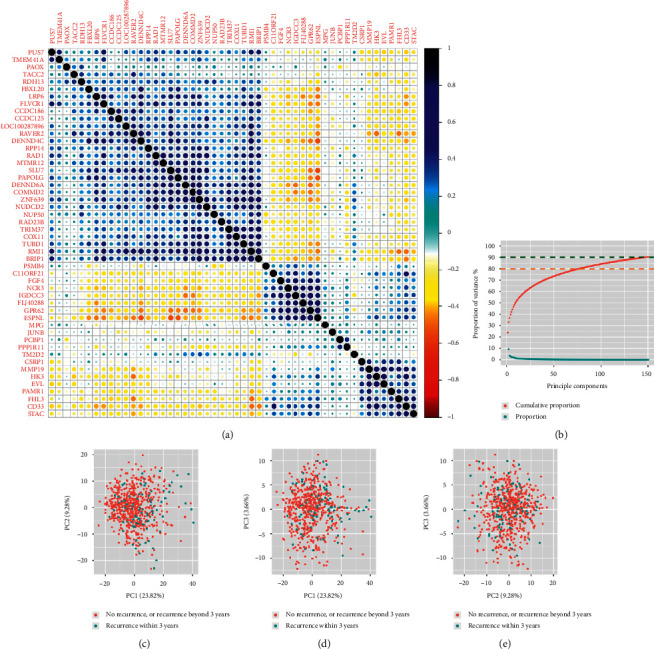
(a) The correlation plot of the randomly selected 50 genes among the 479 universal stage II colorectal cancer recurrence associated genes. The blue color indicates positive correlations, while the red color indicates negative correlations. (b) Proportions of variance explained by principal components. Blue points show the proportion of variance explained by a single principal component, while red points show the cumulative proportions of variance. (c) The scatter plot of the selected principal components (the *x*-axis: PC1; the *y*-axis: PC2). Blue points indicate stage II colorectal cancer patients who had *recurrent events* within 3 years, while red points indicate patients who did not have *recurrent events* within 3 years. (d) The scatter plot of the selected principal components (the *x*-axis: PC1; the *y*-axis: PC3). (e) The scatter plot of the selected principal components (the *x*-axis: PC2; the *y*-axis: PC3).

Before applying machine learning algorithms to predict patients' recurrence risk, we performed correlation analysis and principal component analysis to explore the distribution of gene expression data. As shown in Figure 3(a), we randomly selected 50 genes out of 479 significant genes and calculated their correlation coefficients. Strong correlation of these genes was observed. By performing principal component analysis, we found that the cumulative proportion of variance of the top 77 and 147 principal components reached 80% and 90%, respectively, while the top 3 principal components explained 23.82%, 9.28%, and 3.66% of total variance (Figure 3(b)). Furthermore, the top 3 principal components did not help stratify colorectal cancer recurrence effectively (Figures 3(c)–3(e)). Thus, we did not extract principal components before building predictive models.

### 3.4. Lasso Cox Model

**Figure 4:**
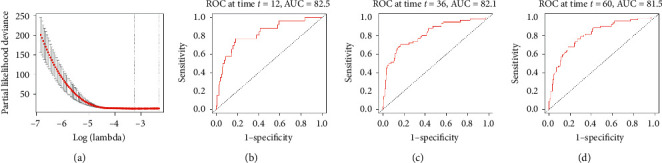
(a) The tuning parameter plot of the lasso Cox model. The *x*-axis represents log-transformed lambda values, and the *y*-axis represents the partial likelihood deviance. The vertical dashed line indicates the minimal partial likelihood deviance. (b) The time-dependent ROC curve of the lasso Cox model at 1 year (12 months), with an AUC of 0.825. (c) The time-dependent ROC curve of the lasso Cox model at 3 years (36 months), with an AUC of 0.821. (d) The time-dependent ROC curve of the lasso Cox model at 5 years (60 months), with an AUC of 0.815.

We randomly divided total samples into the training set (60%) and the test set (40%) and then built the lasso Cox models in the training set. First, the penalty parameter *λ* was determined through 20-fold cross validation to reach the minimal partial likelihood deviance value, and the optimized *λ* value was 0.0387 (Figure 4(a)). The lasso Cox model could reduce the variable dimension by placing a constraint on the absolute value of regression coefficients, so that numerous regression coefficients became smaller or exactly zero [29]. Regression coefficients of 455 genes turned into zero, while the remaining 24 genes were included in the simplified lasso Cox model (Table 2). Eleven regression coefficients of genes were positive, while 13 regression coefficients of genes were negative. Higher expression of genes with positive regression coefficients contributed to higher recurrence risk, while higher expression of genes with negative regression coefficients contributed to lower recurrence risk, respectively. With these regression coefficients, we calculated each patient' recurrence risk in the training set, and then time-dependent ROC curves were plotted. As shown in Figures 4(b)–4(d), AUC values of the lasso Cox model *were* 0.825, 0.821, and 0.815 at 12, 36, and 60 months, respectively. In addition, the Harrell's C-index was 0.805, which implied moderate concordance of the lasso Cox model.

### 3.5. Random Survival Forest Model

**Figure 5:**
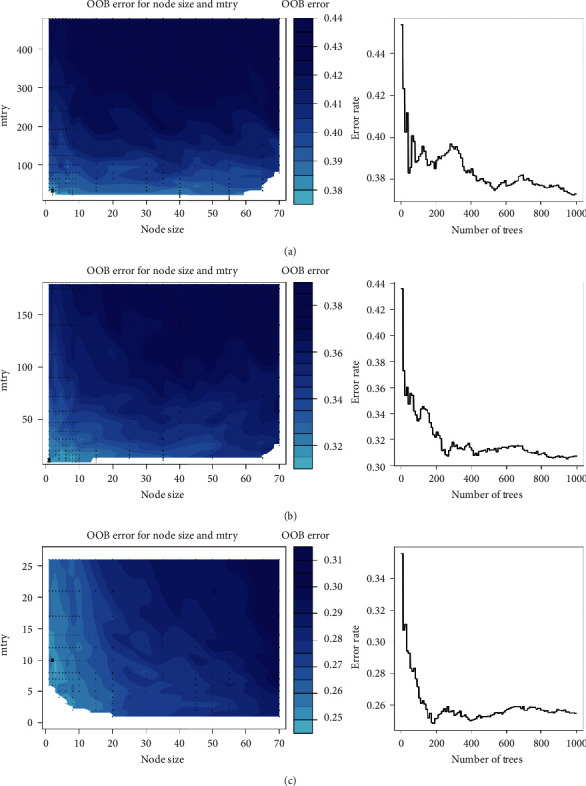
Tuning parameter plots of (a) the primary, (b) the simplified (once), and (c) the simplified (twice) random survival forest model. Left panel: node size is the prespecified number of samples in the terminal node; mtry is the number of candidate variables randomly selected in each parent node; OOB error is the out-of-bag error. A darker color indicates larger OOB error, while a lighter color indicates smaller OOB error. Right panel: the relationship between the number of trees and error rate.

Next, we used the same training set to build and evaluate the random survival forest model. As shown in Figure 5(a), the primary model was based on the total 479 significant genes, and the OOB error was steady when the total number of trees reached 1000. The random survival forest model was an efficient tool for variable selection, and we ranked variables according to the minimal depth of the maximal subtrees and then filtered variables above the cut-off value [30]. In the primary model, there were 300 genes whose minimal depths were above the average cut-off value of 15.06; the remaining 179 genes were fed into the random survival forest model again (Figure 5(b)). Similarly, there were 153 genes above the cut-off value of 11.99 in the simplified (once) model, and the remaining 26 genes were fed into the simplified (twice) model (Figure 5(c)). Finally, all 26 genes were below the cut-off value of 6.35, and model iteration process was successfully completed. The minimal depth of the maximal subtrees of the genes included in the simplified (twice) model is shown in Table 3. The minimal value was 4.39, which was attributed to the NVL gene, and it implied that this gene was the most important in the current model in terms of stratifying the recurrence risk of stage II colorectal cancer patients. It was noteworthy that there were 8 genes (ADNP2, JUNB, MCMBP, FAM46C, NVL, NUP50, JUP, ESM1) included in both models (Figure S3).

**Figure 6:**
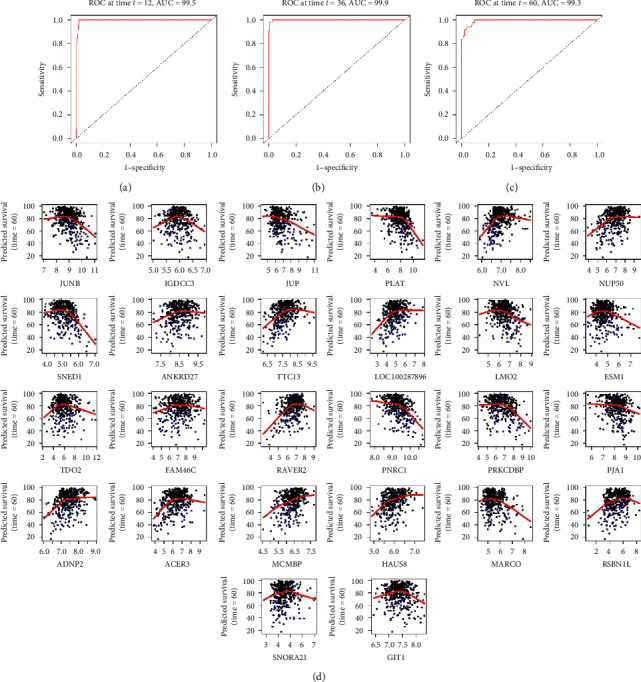
(a) The time-dependent ROC curve of the simplified (twice) random survival forest model at 1 year (12 months), with an AUC of 0.995. (b) The time-dependent ROC curve of the simplified (twice) random survival forest model at 3 years (36 months), with an AUC of 0.999. (c) The time-dependent ROC curve of the simplified (twice) random survival forest model at 5 years (60 months), with an AUC of 0.993. (d) Marginal effects of variables included in the simplified (twice) random survival forest model on the recurrence-free survival probability.

In the simplified (twice) random survival forest model, each patient' recurrence risk in the training set was computed, and then time-dependent ROC curves were plotted. As shown in Figures 6(a)–6(c), AUC values of the models *were* 0.995, 0.999, and 0.993 at 12, 36, and 60 months, respectively. In addition, the Harrell's C-index was 0.986, which implied excellent concordance of the random survival forest model. We also explored marginal effects of gene expression on the recurrence-free survival probability (Figure 6(d)). Using NVL gene as an example, we found that the probability of patients' recurrence-free survival elevated almost linearly when NVL gene expression level increased from 6 to 7, while the probability reached a plateau after its expression level exceeded 7. The majority of the remaining genes had similar trend, yet the expression level of JUP and MCMBP had an *approximately* linear relationship with patients' recurrence-free survival.

**Figure 7:**
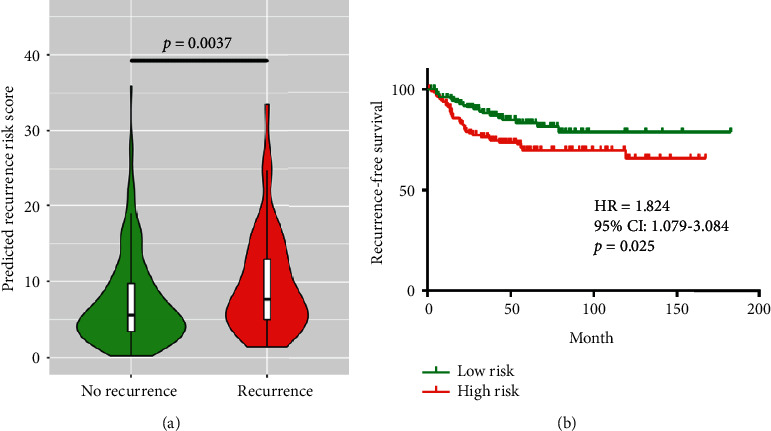
(a) Violin plot of the predicted recurrence risk scores in the test set. Patients who did not experience recurrence are shown in green, and patients who experienced recurrence are shown in red. The Mann–Whitney test was adopted to examine whether predicted recurrence risk scores had statistical significance between groups. (b) Kaplan–Meier survival curve of the random survival forest model prediction results in the test set. Patients who had higher recurrence risk scores than the cut-off value were predicted to be the high risk group (red curve), and patients who had lower recurrence risk scores than the cut-off value were predicted to be the low risk group accordingly (green curve). The log-rank test was performed to find whether survival difference between groups was statistically significant.

We further chose the random survival forest model to evaluate the model performance in the test set, since it had larger AUC value and Harrell's C-index than the lasso Cox model. The expression matrix of the 26 genes *was* imported into the random survival forest model, and recurrence risk score was computed for every *patient* in the test set. As shown in Figure 7(a), we found that the recurrent group had significantly higher recurrence risk scores than the nonrecurrent group (*p* = 0.0037). Using the time-dependent ROC curve of the random survival forest model in the training set, we calculated the optimized cut-off value of recurrence risk score (5.986), under which Youden index reached the maximum. Patients were subsequently divided into the high risk group and the low risk group according to the cut-off value. As shown in Figure 7(b), survival analysis indicated that patients in the high risk group had significantly higher recurrence risk than those in the low risk group (HR = 1.824, 95% CI: 1.079–3.084, *p* = 0.025).

## 4. Discussion

The TNM stage is widely used to predict overall survival and recurrence-free survival of cancer patients; however, the prognosis of colorectal cancer patients in the same stage still varied a lot [38, 39]. Due to the survival heterogeneity of stage II colorectal cancer patients, the prognosis of these patients has drawn many researchers' attention, and they have been trying to discover new prognostic factors in various respects such as pathology, radiology, and biological characteristics of tumor [14, 15]. In the present study, we found prognostic genes that could help stratify stage II colorectal cancer patients' recurrence risk and build predictive models. Different from previous studies [15, 16, 40], we only included stage II colorectal cancer patients from 6 independent microarray datasets, and the total sample size was relatively large. In addition, we performed the microarray meta-analysis to identify universal prognostic genes across datasets, which further increased the robustness of our results, and a total of 479 genes were found to be significantly associated with patients' recurrence-free survival. However, high dimensional data inevitably had the shortcoming of self-correlation to some extent, which was also demonstrated by the correlation analysis in our study. Furthermore, a model requiring the expression of hundreds of genes was not appropriate for clinical practice. Therefore, it was crucial to perform feature selection and model simplification.

Most of the previous studies adopted the Cox proportional hazard model with lasso penalization for high dimensional data [15, 41], yet the Cox model made an assumption that the impact of variables on survival risk was linear. Furthermore, previous studies did not compare the performance of other models with the lasso Cox model. Unlike recently emerging classification models, there were not too many survival prediction models available; however, the random survival forest model has been gradually utilized by researchers for survival prediction. For instance, Paik et al. developed a 21-gene-based random survival forest model to predict progression-free survival of ovarian cancer [42]. Jung et al. identified insulin resistance SNPs in combination with lifestyle factors for breast cancer risk prediction via the random survival forest model [43]. In the current study, the random survival forest model had larger AUC and Harrell's C-index than *the* lasso Cox model. By performing marginal effects analysis, we also found the nonlinear relationship between gene expression value and patients' recurrence-free survival probability. In addition, several genes included in both models, such as JUP and ESM1, were reported to be associated with the prognosis of colorectal cancer patients by other researchers [44, 45], which proved that the results of both models were reliable.

However, the current study was inevitably limited in some aspects. First, we only included the gene expression data from public databases, and the demographic and clinicopathological characteristics were not available. We believed that the performance of our model will be further improved with features such as tumor differentiation and MMR status. Second, although our model was based on the microarray meta-analysis, it still needs to be validated by large-scale prospective cohort studies involving stage II colorectal cancer patients. We did not further perform multiple platform analysis such as RNA-Seq or immunohistochemistry, since the sample sizes of public datasets which specifically included stage II colorectal cancer patients were relatively small, and many of them did not provide recurrence-free survival follow-up information. In addition, the molecular mechanisms of these genes were unknown, and a group of dysregulated genes rather than certain single gene may participate in the recurrence of stage II colorectal cancer. In the future, more mechanistic studies and multiomics studies will be needed to address how these genes contributed to stage II colorectal cancer recurrence.

## 5. Conclusions

We identified 479 stage II colorectal cancer recurrence associated genes by microarray meta-analysis. Enrichment analysis indicated that G protein-coupled glutamate receptor binding and Hedgehog signaling may be associated with colorectal cancer recurrence. Two survival models with feature selection process were trained, and the random survival forest model outperformed the linear lasso Cox model. Based on the risk score, the random survival forest model could strongly predict the recurrence risk of stage II colorectal cancer patients.

## Figures and Tables

**Table 1:** Characteristics of the included datasets.

Dataset accession	Microarray platform	Sample size	RFS follow-up months	Recurrence event
GSE14333	GPL570	94	2.26–118.58 (median: 37.66)	14/94
GSE17538	GPL570	70	0.43–118.58 (median: 37.31)	11/70
GSE33113	GPL570	89	1.8–119.97 (median: 39.47)	18/91
GSE39582	GPL570	267	0–201 (median: 53)	61/267
GSE92921	GPL570	43	6.4–139.7 (median: 74.63)	2/43
GSE24551	GPL5175	88	5.04–120 (median: 47.82)	27/90

**Table 2:** Regression coefficients of the lasso Cox model.

Gene symbol	*β* ^*∗*^	Gene symbol	*β* ^*∗*^
PAOX	−0.02752	FLJ90680	0.011181
SIGLEC7	0.174815	NVL	−0.0407
PHAX	−0.02803	ESM1	0.158185
XCR1	0.073122	GABRR2	0.055987
TM4SF4	0.021224	FAM166A	−0.56059
TRIOBP	0.173985	USP14	−0.03345
MCMBP	−0.10873	JUNB	0.268267
HCFC1R1	0.029341	UBAP2	−0.41623
ADNP2	−0.1739	AP5B1	−0.3134
NUP50	−0.02431	FAM46C	−0.02913
GTF2A2	−0.01368	LDB3	0.146108
BCCIP	−0.03355	JUP	0.260895

^*∗*^Positive regression coefficients indicate that higher gene expression values contributed to higher recurrence risks, while negative coefficients indicate that higher gene expression values contributed to lower recurrence risks.

**Table 3:** Minimal depths of variables in the simplified (twice) random survival forest model.

Gene symbol	Minimal depths^*∗*^ (threshold = 6.35)	Gene symbol	Minimal depths^*∗*^ (threshold = 6.35)
NVL	4.39	SNED1	5.553
ACER3	4.719	ESM1	5.59
JUP	4.816	MARCO	5.599
PLAT	4.834	FAM46C	5.744
JUNB	4.913	LMO2	5.772
IGDCC3	4.965	HAUS8	5.791
ANKRD27	5.029	TTC13	5.823
NUP50	5.09	ADNP2	5.843
GIT1	5.244	RSBN1L	5.863
PRKCDBP	5.321	RAVER2	5.883
TDO2	5.349	SNORA21	6.054
LOC100287896	5.397	PNRC1	6.205
MCMBP	5.498	PJA1	6.338

^*∗*^Minimal depths of all variables in the simplified (twice) random survival forest model were under the threshold.

## Data Availability

All the data generated or analyzed during this study are included within this article.

## Abbreviations

AUC: Area under the receiver operating characteristic curve
FDR: False discovery rate
GO: Gene ontology
HR: Hazard ratio
CI: Confidence interval
KEGG: Kyoto encyclopedia of genes and genomes
OOB: Out-of-bag
ROC: Receiver operating characteristic
MMR: Mismatch repair.

## Supplementary Materials

Supplementary Materials Figure S1: schematic diagram of random survival forest model. Figure S2: protein-protein interaction network of the selected 479 enes. Figure S3: Venn diagram of the included genes in both models. Table S1: common stage II colorectal cancer recurrence associated genes.
