# Supplementary material for: Identifying Stage II Colorectal Cancer Recurrence Associated Genes by Microarray Meta-Analysis and Building Predictive Models with Machine Learning Algorithms
Source: J Oncol. 2021 Feb 10;2021:6657397. doi: 10.1155/2021/6657397 (PMC7889382; doi:10.1155/2021/6657397)
Supplement: Supplementary Materials — Figure S1: schematic diagram of random survival forest model. Figure S2: protein-protein interaction network of the selected 479 enes. Figure S3: Venn diagram of the included genes in both models. Table S1: common stage II colorectal cancer recurrence associated genes. [file 6657397.f1.docx]

**
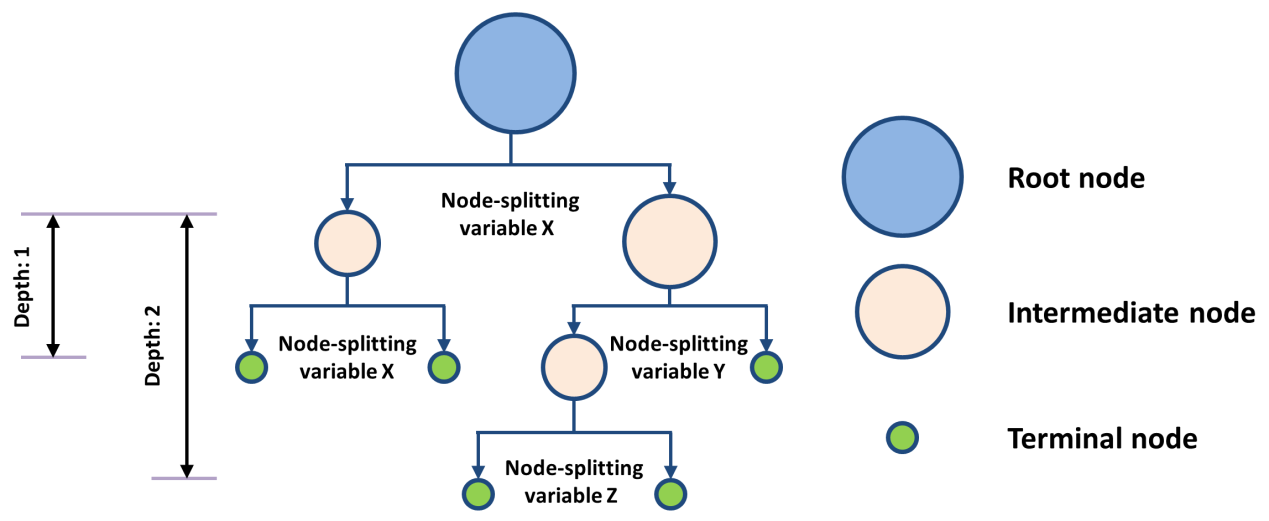
**

**Figure S1. Schematic diagram of random survival forest model.** Each survival tree had one root node that would branch out into two child nodes. A given number of variables were randomly selected as candidate variables in each root node, then the optimized cut-off values of candidate variables were generated to maximize the survival difference of child nodes, and only the most discriminative candidate variable would become the node-splitting variable. Total samples were divided into 2 groups according to the node-splitting variable. Iteratively, intermediate child nodes became parent nodes and the above node-splitting processes were repeated until the node size was less than the pre-specified number. Minimal depths of variables reflected the priority of being selected as node-splitting variables, and smaller minimal depths indicated greater importance of variables. The minimal depth of the variable which splitted the root node was defined as 0, and the minimal depths of other variables were the nearest distance from the current nodes to the root node. For example, the minimal depths of variable X, Y and Z were 0, 1 and 2.


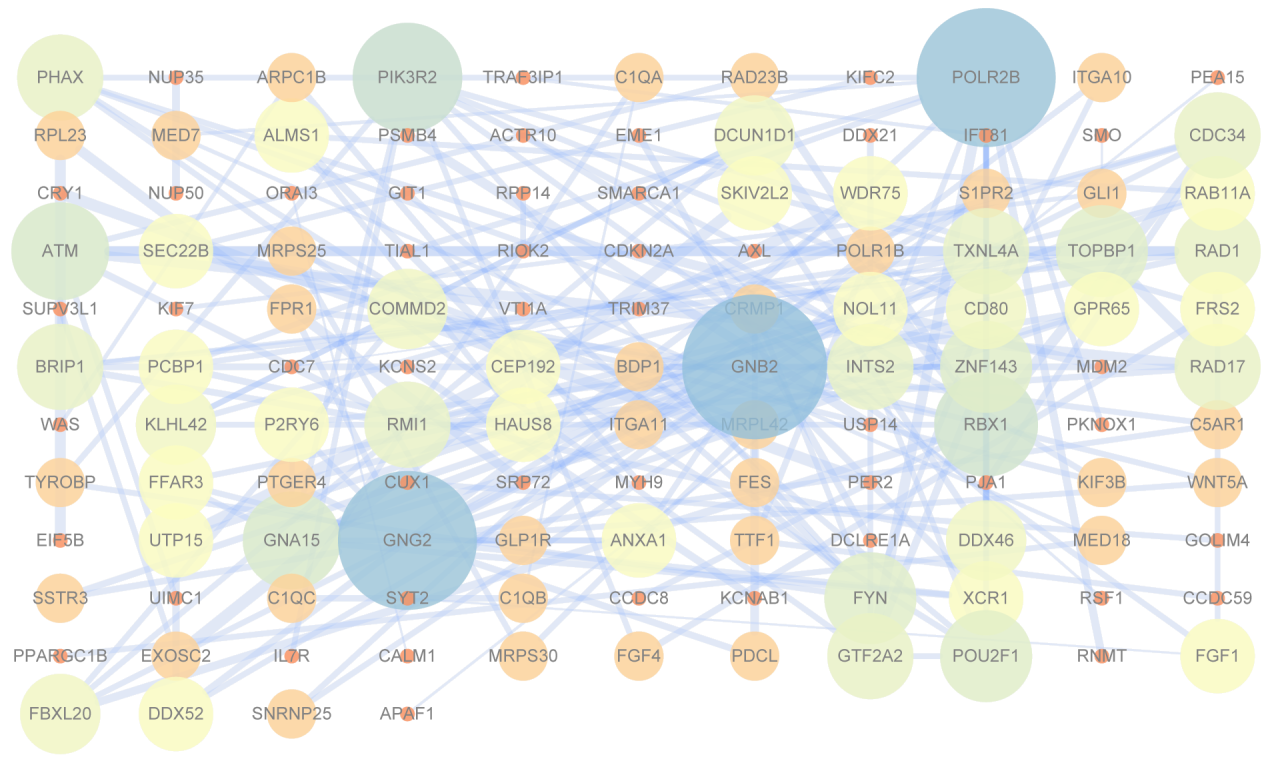


**Figure S2. Protein-protein interaction network of the selected 479 genes.** The sizes of circles were proportional to the number of interacting proteins, and the thickness of lines were proportional to the predcicted interacting strength.


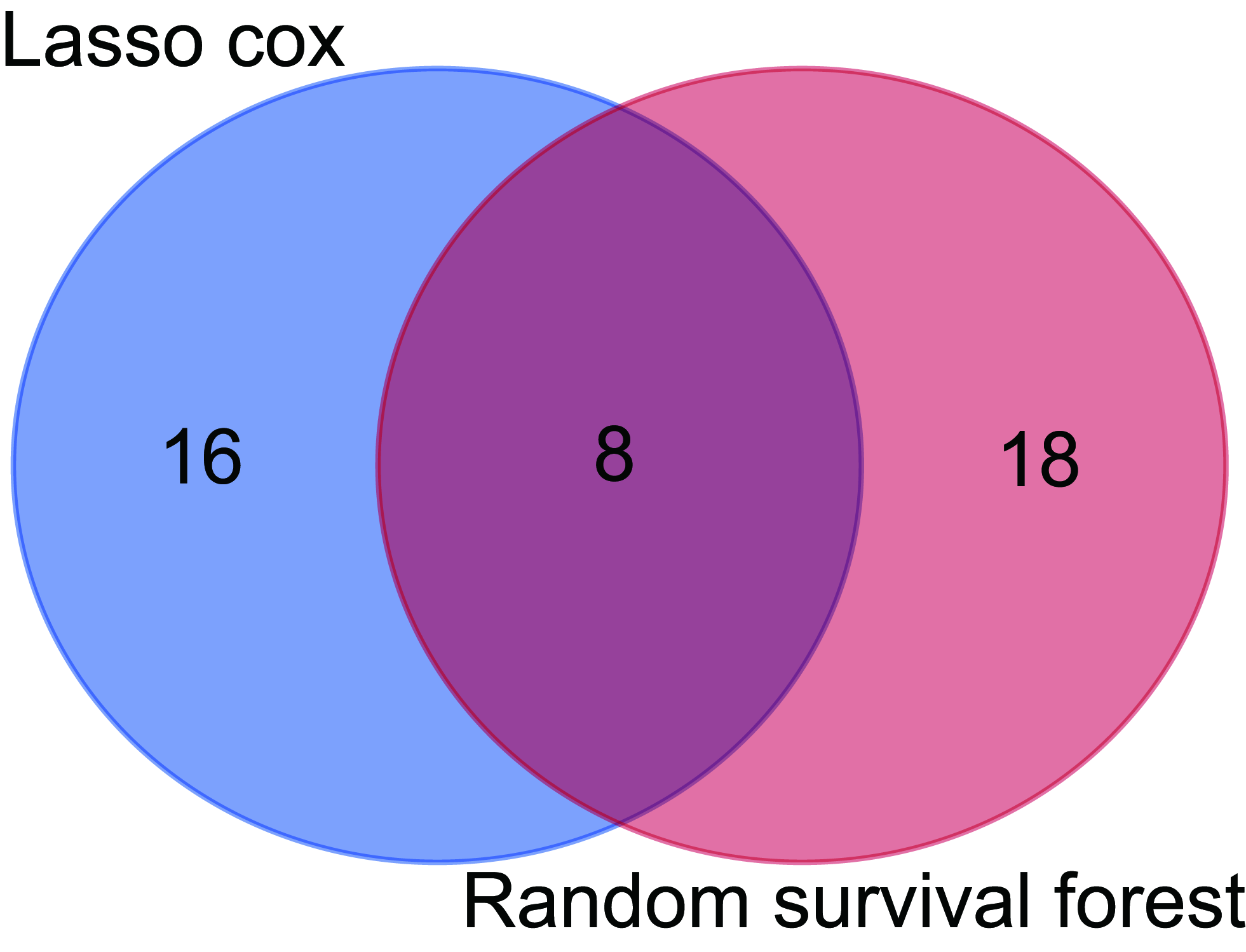


**Figure S3. Venn diagram of the included genes in both models.**

| **Table S1. Common stage II colorectal cancer recurrence associated genes** | | | | | | | | |
| --- | --- | --- | --- | --- | --- | --- | --- | --- |
| Rank | Gene symbol | FDR | Rank | Gene symbol | FDR | Rank | Gene symbol | FDR |
| 1 | FGF4 | 0.006 | 41 | ALMS1 | 0.034 | 81 | HSD17B14 | 0.047 |
| 2 | ZDHHC23 | 0.008 | 42 | PRPH2 | 0.034 | 82 | ARMCX6 | 0.048 |
| 3 | PPP1R11 | 0.014 | 43 | CLDN6 | 0.035 | 83 | MTMR12 | 0.048 |
| 4 | FGD2 | 0.014 | 44 | CD300C | 0.035 | 84 | COX11 | 0.048 |
| 5 | TRIM33 | 0.014 | 45 | RHOJ | 0.035 | 85 | JMY | 0.048 |
| 6 | ADAP2 | 0.014 | 46 | WAS | 0.035 | 86 | FEZ1 | 0.048 |
| 7 | EPOR | 0.014 | 47 | GPR65 | 0.035 | 87 | WDR45B | 0.050 |
| 8 | OTULIN | 0.014 | 48 | NHLRC2 | 0.035 | 88 | GCC2-AS1 | 0.050 |
| 9 | CTF1 | 0.014 | 49 | VTI1A | 0.036 | 89 | DNAJB5 | 0.050 |
| 10 | RETN | 0.014 | 50 | R3HDML | 0.036 | 90 | GEN1 | 0.050 |
| 11 | CHI3L2 | 0.014 | 51 | KLHL42 | 0.036 | 91 | WNT5A | 0.050 |
| 12 | COA7 | 0.014 | 52 | SIRT2 | 0.037 | 92 | ZNF143 | 0.050 |
| 13 | ERCC6L2 | 0.014 | 53 | HAUS8 | 0.037 | 93 | HOOK1 | 0.050 |
| 14 | DDX52 | 0.014 | 54 | TOPBP1 | 0.039 | 94 | SYNPO2L | 0.050 |
| 15 | ADAM23 | 0.015 | 55 | BPGM | 0.039 | 95 | TIAL1 | 0.050 |
| 16 | PNRC1 | 0.019 | 56 | RAPGEF6 | 0.039 | 96 | LRRN4CL | 0.052 |
| 17 | RMI1 | 0.019 | 57 | SCAI | 0.039 | 97 | BRWD1 | 0.052 |
| 18 | KRT81 | 0.019 | 58 | SHTN1 | 0.043 | 98 | ZNF507 | 0.052 |
| 19 | C11ORF21 | 0.021 | 59 | DENND4C | 0.043 | 99 | SHQ1 | 0.052 |
| 20 | TDO2 | 0.023 | 60 | PHAX | 0.043 | 100 | SYT2 | 0.052 |
| 21 | CTSB | 0.024 | 61 | GMCL1 | 0.043 | 101 | GTF3C4 | 0.053 |
| 22 | PPARGC1B | 0.024 | 62 | DNAJC24 | 0.043 | 102 | DNAJC9 | 0.054 |
| 23 | POU2F1 | 0.024 | 63 | CLSTN3 | 0.043 | 103 | PCBP1 | 0.054 |
| 24 | ESPNL | 0.024 | 64 | TMEM59L | 0.043 | 104 | LYRM7 | 0.054 |
| 25 | GLP1R | 0.024 | 65 | TM4SF18 | 0.043 | 105 | ECE1 | 0.055 |
| 26 | FGF1 | 0.024 | 66 | CHST1 | 0.043 | 106 | SLC35A3 | 0.055 |
| 27 | PAOX | 0.024 | 67 | FCN1 | 0.043 | 107 | TRIOBP | 0.055 |
| 28 | SLF1 | 0.024 | 68 | HIST1H2AK | 0.043 | 108 | TTF1 | 0.055 |
| 29 | KIF7 | 0.024 | 69 | IGFL1 | 0.043 | 109 | FAM204A | 0.055 |
| 30 | SNCA | 0.030 | 70 | XCR1 | 0.043 | 110 | LRIG2 | 0.055 |
| 31 | INTS2 | 0.032 | 71 | CLEC4F | 0.043 | 111 | IQCJ-SCHIP1 | 0.055 |
| 32 | METTL8 | 0.032 | 72 | NCR3 | 0.043 | 112 | FAM110D | 0.055 |
| 33 | OR1I1 | 0.032 | 73 | RBM15 | 0.043 | 113 | CSRP1 | 0.056 |
| 34 | CD80 | 0.032 | 74 | EI24 | 0.047 | 114 | SEC22B | 0.056 |
| 35 | IGFBP3 | 0.033 | 75 | NOL7 | 0.047 | 115 | C5ORF51 | 0.056 |
| 36 | NAA25 | 0.033 | 76 | UBA5 | 0.047 | 116 | ZBTB21 | 0.056 |
| 37 | RSF1 | 0.033 | 77 | MED7 | 0.047 | 117 | FPR1 | 0.056 |
| 38 | SIGLEC7 | 0.033 | 78 | ARHGAP32 | 0.047 | 118 | KCNS2 | 0.056 |
| 39 | NPR2 | 0.033 | 79 | RAD1 | 0.047 | 119 | LRRC71 | 0.056 |
| 40 | ZBTB44 | 0.034 | 80 | TM4SF4 | 0.047 | 120 | MCMBP | 0.057 |

| Rank | Gene symbol | FDR | Rank | Gene symbol | FDR | Rank | Gene symbol | FDR |
| --- | --- | --- | --- | --- | --- | --- | --- | --- |
| 121 | C10ORF12 | 0.057 | 161 | IGDCC3 | 0.060 | 201 | SNORA21 | 0.063 |
| 122 | VSIG4 | 0.057 | 162 | MCEMP1 | 0.060 | 202 | TMEM108 | 0.063 |
| 123 | MPHOSPH9 | 0.057 | 163 | ZFYVE1 | 0.060 | 203 | ZNF564 | 0.064 |
| 124 | ARPC1B | 0.057 | 164 | GJB4 | 0.060 | 204 | HOMER3 | 0.064 |
| 125 | LMCD1 | 0.057 | 165 | LOC100287896 | 0.060 | 205 | CDC34 | 0.064 |
| 126 | PIP4K2A | 0.057 | 166 | SHISA4 | 0.060 | 206 | UBTD2 | 0.064 |
| 127 | HCFC1R1 | 0.057 | 167 | SCARA3 | 0.060 | 207 | CEP95 | 0.064 |
| 128 | ITGA11 | 0.057 | 168 | DYRK1A | 0.061 | 208 | SLC22A5 | 0.065 |
| 129 | KIFC2 | 0.057 | 169 | NSRP1 | 0.061 | 209 | TACC2 | 0.066 |
| 130 | PRKAG3 | 0.058 | 170 | FAM214B | 0.061 | 210 | BCCIP | 0.066 |
| 131 | LRRC8B | 0.059 | 171 | SUV39H2 | 0.061 | 211 | CEP192 | 0.067 |
| 132 | ADAMTS5 | 0.059 | 172 | C6ORF15 | 0.061 | 212 | ORAI3 | 0.069 |
| 133 | OLAH | 0.059 | 173 | PROCA1 | 0.061 | 213 | CCDC43 | 0.069 |
| 134 | GNG2 | 0.059 | 174 | C1QB | 0.062 | 214 | UIMC1 | 0.069 |
| 135 | IGFBP7 | 0.060 | 175 | RBPMS | 0.062 | 215 | CCDC186 | 0.069 |
| 136 | GNB2 | 0.060 | 176 | GNA15 | 0.062 | 216 | DOK2 | 0.069 |
| 137 | CYB5R3 | 0.060 | 177 | GPR62 | 0.062 | 217 | HK3 | 0.069 |
| 138 | LMNA | 0.060 | 178 | LURAP1 | 0.062 | 218 | RSBN1L | 0.069 |
| 139 | NOL11 | 0.060 | 179 | BEST1 | 0.062 | 219 | STAC | 0.069 |
| 140 | RAD23B | 0.060 | 180 | BRIP1 | 0.062 | 220 | C1ORF52 | 0.069 |
| 141 | ASCL2 | 0.060 | 181 | SDHC | 0.062 | 221 | CCDC8 | 0.069 |
| 142 | RGS2 | 0.060 | 182 | RAVER2 | 0.063 | 222 | GAB3 | 0.069 |
| 143 | PDZD8 | 0.060 | 183 | TUSC3 | 0.063 | 223 | BST1 | 0.069 |
| 144 | PJA1 | 0.060 | 184 | MKLN1 | 0.063 | 224 | ZNF235 | 0.069 |
| 145 | GPBP1 | 0.060 | 185 | RNF103 | 0.063 | 225 | TEX37 | 0.069 |
| 146 | PLD3 | 0.060 | 186 | MPG | 0.063 | 226 | FLJ90680 | 0.069 |
| 147 | KLHL8 | 0.060 | 187 | ADNP2 | 0.063 | 227 | CXORF36 | 0.069 |
| 148 | BAG1 | 0.060 | 188 | NUP50 | 0.063 | 228 | BPIFA4P | 0.069 |
| 149 | TTC13 | 0.060 | 189 | FBXO33 | 0.063 | 229 | APOBEC3A_B | 0.069 |
| 150 | COMMD2 | 0.060 | 190 | PRR14L | 0.063 | 230 | MIR31HG | 0.069 |
| 151 | TET3 | 0.060 | 191 | TMEM68 | 0.063 | 231 | ADGRG3 | 0.069 |
| 152 | TRIM2 | 0.060 | 192 | GAL3ST4 | 0.063 | 232 | ATP1B2 | 0.069 |
| 153 | TMEM209 | 0.060 | 193 | FOLR2 | 0.063 | 233 | PTPRT | 0.069 |
| 154 | FLVCR1 | 0.060 | 194 | GTF2A2 | 0.063 | 234 | SSC4D | 0.069 |
| 155 | LIG3 | 0.060 | 195 | PRDM10 | 0.063 | 235 | PCNX1 | 0.069 |
| 156 | MRPL42 | 0.060 | 196 | TMEM268 | 0.063 | 236 | SNRNP25 | 0.069 |
| 157 | PRRX2 | 0.060 | 197 | GK5 | 0.063 | 237 | EXOSC2 | 0.069 |
| 158 | ZBTB2 | 0.060 | 198 | ZNF565 | 0.063 | 238 | CSF1R | 0.069 |
| 159 | ARHGAP24 | 0.060 | 199 | GLI1 | 0.063 | 239 | SSX2IP | 0.069 |
| 160 | CALM1 | 0.060 | 200 | CDKN2A | 0.063 | 240 | BDP1 | 0.069 |

| Rank | Gene symbol | FDR | Rank | Gene symbol | FDR | Rank | Gene symbol | FDR |
| --- | --- | --- | --- | --- | --- | --- | --- | --- |
| 241 | RPP14 | 0.069 | 281 | STXBP6 | 0.073 | 321 | RALBP1 | 0.079 |
| 242 | CLEC4A | 0.069 | 282 | MNDA | 0.073 | 322 | ACER3 | 0.079 |
| 243 | FES | 0.069 | 283 | RNMT | 0.073 | 323 | MMP19 | 0.079 |
| 244 | M1AP | 0.069 | 284 | TYROBP | 0.074 | 324 | TMPRSS6 | 0.079 |
| 245 | ADAMTS16 | 0.069 | 285 | OXNAD1 | 0.074 | 325 | PEA15 | 0.080 |
| 246 | PSMB4 | 0.069 | 286 | GOLIM4 | 0.074 | 326 | FFAR3 | 0.080 |
| 247 | FNDC4 | 0.069 | 287 | SSBP3 | 0.074 | 327 | CREM | 0.080 |
| 248 | EME1 | 0.069 | 288 | PLB1 | 0.074 | 328 | USP25 | 0.080 |
| 249 | C1QC | 0.070 | 289 | THAP12 | 0.074 | 329 | FLCN | 0.081 |
| 250 | NVL | 0.070 | 290 | NUP35 | 0.074 | 330 | MYH9 | 0.082 |
| 251 | ATXN2L | 0.070 | 291 | ATM | 0.074 | 331 | MXRA8 | 0.082 |
| 252 | ANKRD27 | 0.070 | 292 | USP12 | 0.074 | 332 | GTSF1 | 0.082 |
| 253 | RBM27 | 0.070 | 293 | C19ORF24 | 0.075 | 333 | CLDN5 | 0.082 |
| 254 | FRS2 | 0.070 | 294 | PAPOLG | 0.075 | 334 | ESCO1 | 0.082 |
| 255 | KLRG1 | 0.070 | 295 | FOSL1 | 0.075 | 335 | RASAL3 | 0.082 |
| 256 | FLJ11710 | 0.070 | 296 | PHC3 | 0.075 | 336 | PSMC3IP | 0.082 |
| 257 | PRELID2 | 0.070 | 297 | GABRR2 | 0.075 | 337 | CFL2 | 0.082 |
| 258 | DCLRE1A | 0.070 | 298 | FAM166A | 0.075 | 338 | TM2D2 | 0.084 |
| 259 | PTGER4 | 0.071 | 299 | SERPINA3 | 0.075 | 339 | PER2 | 0.086 |
| 260 | FUNDC2 | 0.071 | 300 | PLAT | 0.076 | 340 | TXNL4A | 0.086 |
| 261 | EIF3A | 0.071 | 301 | GIT1 | 0.076 | 341 | CXORF56 | 0.086 |
| 262 | CRY1 | 0.071 | 302 | UBLCP1 | 0.076 | 342 | USP14 | 0.087 |
| 263 | DDX46 | 0.071 | 303 | HSF4 | 0.076 | 343 | DENND6A | 0.087 |
| 264 | PDE4A | 0.071 | 304 | AADAT | 0.077 | 344 | PUS7 | 0.087 |
| 265 | APCDD1L | 0.071 | 305 | FBXL20 | 0.077 | 345 | CCDC59 | 0.087 |
| 266 | ITGA10 | 0.071 | 306 | BRAP | 0.077 | 346 | LRP6 | 0.087 |
| 267 | SUPV3L1 | 0.071 | 307 | DDX21 | 0.078 | 347 | AXL | 0.087 |
| 268 | CCNJ | 0.071 | 308 | KCNIP3 | 0.078 | 348 | EXOC3L2 | 0.087 |
| 269 | CCDC125 | 0.071 | 309 | TMEM256-PLSCR3 | 0.079 | 349 | GABARAPL1 | 0.087 |
| 270 | C1QA | 0.072 | 310 | PACS1 | 0.079 | 350 | PRKCDBP | 0.087 |
| 271 | LMO2 | 0.072 | 311 | SRP72 | 0.079 | 351 | CCDC84 | 0.087 |
| 272 | MARCO | 0.072 | 312 | VIM | 0.079 | 352 | CYP2R1 | 0.087 |
| 273 | EVL | 0.072 | 313 | KLF2 | 0.079 | 353 | GRASP | 0.087 |
| 274 | UTP15 | 0.072 | 314 | FBRSL1 | 0.079 | 354 | IFT81 | 0.087 |
| 275 | RBX1 | 0.073 | 315 | MDM2 | 0.079 | 355 | EMSY | 0.087 |
| 276 | SKIV2L2 | 0.073 | 316 | IL7R | 0.079 | 356 | NFAM1 | 0.087 |
| 277 | TMEM161B | 0.073 | 317 | NUDCD2 | 0.079 | 357 | FLJ40288 | 0.087 |
| 278 | KLHDC8B | 0.073 | 318 | CPA4 | 0.079 | 358 | SMO | 0.087 |
| 279 | PKNOX1 | 0.073 | 319 | MYOG | 0.079 | 359 | MAPK8IP2 | 0.087 |
| 280 | ESM1 | 0.073 | 320 | DNAJC5B | 0.079 | 360 | EIF5B | 0.087 |

| Rank | Gene symbol | FDR | Rank | Gene symbol | FDR | Rank | Gene symbol | FDR |
| --- | --- | --- | --- | --- | --- | --- | --- | --- |
| 361 | C5ORF46 | 0.087 | 401 | ETAA1 | 0.091 | 441 | SLC6A2 | 0.094 |
| 362 | ADGRV1 | 0.087 | 402 | INE1 | 0.091 | 442 | RDH13 | 0.094 |
| 363 | JUNB | 0.087 | 403 | FAM46C | 0.092 | 443 | TTC17 | 0.095 |
| 364 | UBAP2 | 0.087 | 404 | ANXA1 | 0.092 | 444 | WDR27 | 0.095 |
| 365 | ARHGAP8 | 0.087 | 405 | ARHGAP33 | 0.093 | 445 | PINK1-AS | 0.095 |
| 366 | ZNF271P | 0.087 | 406 | TMEM41A | 0.093 | 446 | HS3ST3B1 | 0.095 |
| 367 | MRPS30 | 0.088 | 407 | GPSM3 | 0.093 | 447 | NOC3L | 0.095 |
| 368 | POLR2B | 0.088 | 408 | LYST | 0.093 | 448 | SNED1 | 0.095 |
| 369 | PAXBP1 | 0.088 | 409 | RIOK2 | 0.093 | 449 | ADAMTS4 | 0.095 |
| 370 | AGGF1 | 0.088 | 410 | LDB3 | 0.093 | 450 | CD40LG | 0.095 |
| 371 | DNAJC22 | 0.088 | 411 | PI15 | 0.093 | 451 | MAB21L2 | 0.096 |
| 372 | C9ORF85 | 0.088 | 412 | MED18 | 0.093 | 452 | ACTR10 | 0.096 |
| 373 | SSTR3 | 0.088 | 413 | PARK7 | 0.093 | 453 | KIF3B | 0.096 |
| 374 | AP5B1 | 0.088 | 414 | AK3 | 0.093 | 454 | DCUN1D1 | 0.097 |
| 375 | RASGRP3 | 0.089 | 415 | NEK4 | 0.093 | 455 | JUP | 0.097 |
| 376 | CD14 | 0.089 | 416 | SNX11 | 0.093 | 456 | HIC1 | 0.097 |
| 377 | PRAF2 | 0.089 | 417 | SLC44A1 | 0.093 | 457 | TAMM41 | 0.097 |
| 378 | MARCH5 | 0.089 | 418 | ZNF582 | 0.093 | 458 | RNASE3 | 0.097 |
| 379 | C5AR1 | 0.089 | 419 | CD33 | 0.093 | 459 | OR7C2 | 0.097 |
| 380 | GXYLT1 | 0.089 | 420 | SYNDIG1 | 0.093 | 460 | SOX15 | 0.097 |
| 381 | S1PR2 | 0.089 | 421 | ADAMTS10 | 0.093 | 461 | PAIP1 | 0.097 |
| 382 | NTN1 | 0.089 | 422 | SLU7 | 0.094 | 462 | TGM2 | 0.097 |
| 383 | PPP4R2 | 0.089 | 423 | PAQR3 | 0.094 | 463 | PALMD | 0.097 |
| 384 | IGFBP6 | 0.089 | 424 | ILF3-AS1 | 0.094 | 464 | LSP1 | 0.097 |
| 385 | ZNF639 | 0.089 | 425 | TROVE2 | 0.094 | 465 | ROGDI | 0.097 |
| 386 | PAX8 | 0.089 | 426 | GPAM | 0.094 | 466 | TRIM37 | 0.097 |
| 387 | FYN | 0.091 | 427 | CCM2L | 0.094 | 467 | FHL3 | 0.098 |
| 388 | ZDHHC8 | 0.091 | 428 | FAM206A | 0.094 | 468 | ZCCHC10 | 0.098 |
| 389 | TRAPPC13 | 0.091 | 429 | AIF1 | 0.094 | 469 | MRPS25 | 0.098 |
| 390 | RAB11A | 0.091 | 430 | ECM1 | 0.094 | 470 | KCNAB1 | 0.098 |
| 391 | ALDH5A1 | 0.091 | 431 | TSC1 | 0.094 | 471 | SMARCA1 | 0.099 |
| 392 | WDR75 | 0.091 | 432 | PIK3R2 | 0.094 | 472 | POLR1B | 0.099 |
| 393 | PDCL | 0.091 | 433 | TRAF3IP1 | 0.094 | 473 | PLPPR2 | 0.099 |
| 394 | APAF1 | 0.091 | 434 | CDC7 | 0.094 | 474 | N4BP2 | 0.099 |
| 395 | MOCS2 | 0.091 | 435 | PPHLN1 | 0.094 | 475 | PLA2G3 | 0.099 |
| 396 | MZF1 | 0.091 | 436 | ZMYM1 | 0.094 | 476 | ARID5A | 0.099 |
| 397 | SMAD5 | 0.091 | 437 | P2RY6 | 0.094 | 477 | RAD17 | 0.100 |
| 398 | SLC25A14 | 0.091 | 438 | TUBD1 | 0.094 | 478 | CRMP1 | 0.100 |
| 399 | NCALD | 0.091 | 439 | IL1RL1 | 0.094 | 479 | SBNO1 | 0.100 |
| 400 | PAMR1 | 0.091 | 440 | ASTN2 | 0.094 |  |  |  |

FDR: false discovery rate.
